# Reimagining Human Research Protections for 21st Century Science

**DOI:** 10.2196/jmir.6634

**Published:** 2016-12-22

**Authors:** Cinnamon Bloss, Camille Nebeker, Matthew Bietz, Deborah Bae, Barbara Bigby, Mary Devereaux, James Fowler, Ann Waldo, Nadir Weibel, Kevin Patrick, Scott Klemmer, Lori Melichar

**Affiliations:** ^1^ Center for Wireless and Population Health Systems, The Qualcomm Institute, Calit2 University of California, San Diego La Jolla, CA United States; ^2^ Department of Psychiatry University of California, San Diego La Jolla, CA United States; ^3^ Department of Family Medicine and Public Health, School of Medicine University of California, San Diego La Jolla, CA United States; ^4^ Department of Informatics Donald Bren School of Information and Computer Sciences University of California, Irvine Irvine, CA United States; ^5^ Robert Wood Johnson Foundation Princeton, NJ United States; ^6^ Scripps Human Research Protections Program La Jolla, CA United States; ^7^ Department of Pathology, School of Medicine University of California, San Diego La Jolla, CA United States; ^8^ Department of Political Science University of California, San Diego La Jolla, CA United States; ^9^ Department of Medicine School of Medicine University of California, San Diego La Jolla, CA United States; ^10^ Waldo Law Offices, PLLC Washington, DC United States; ^11^ Department of Computer Science and Engineering University of California, San Diego La Jolla, CA United States; ^12^ Department of Cognitive Science University of California, San Diego La Jolla, CA United States

**Keywords:** ethics committees, research, biomedical research, telemedicine, informed consent, behavioral research

## Abstract

**Background:**

Evolving research practices and new forms of research enabled by technological advances require a redesigned research oversight system that respects and protects human research participants.

**Objective:**

Our objective was to generate creative ideas for redesigning our current human research oversight system.

**Methods:**

A total of 11 researchers and institutional review board (IRB) professionals participated in a January 2015 design thinking workshop to develop ideas for redesigning the IRB system.

**Results:**

Ideas in 5 major domains were generated. The areas of focus were (1) improving the consent form and process, (2) empowering researchers to protect their participants, (3) creating a system to learn from mistakes, (4) improving IRB efficiency, and (5) facilitating review of research that leverages technological advances.

**Conclusions:**

We describe the impetus for and results of a design thinking workshop to reimagine a human research protections system that is responsive to 21st century science.

## Introduction

Over half a century ago, in response to egregious cases of research participant mistreatment, the US government proposed prospective review of research involving human participants. This prospective review process is what we now know as the institutional review board (IRB) system. Today, IRBs are firmly entrenched within the fabric of academic research institution, with estimates putting the number of IRBs in the United States at around 6000 (I Prichard, Senior Advisor to the Director of the Office for Human Research Protections, Department of Health and Human Services; oral communication, September 2014).

IRBs have aimed to serve an important function, which is to protect human research participants. While IRBs have helped address this critical need, the IRB system has not kept pace with the evolution of research methods and practices or current and emerging trends in science and technology. The fact that the system has become antiquated calls into question whether the IRB continues to foster the protection of human research participants per the principles originally put forth in the Belmont Report [1]. New forms of research enabled by technological advances in information technology and data science appear to be particularly challenging to IRBs [2], yet clear standards to guide best practices are not well established [3-5]. We propose that the time has come to reimagine and ultimately work toward redesigning our human research protections system so that it is responsive to both the evolution of general research practices and new forms of research enabled by technological advances—what we refer to here as 21st century science. This is critical for the proper protection of research participants, ethical and efficient use of research resources, and facilitation of research insights important for human health specifically and knowledge production more generally.

### A Changing Research Landscape

The IRB model was created when research was typically conducted by a single principal investigator in a single academic institution, and when data were both scarce and expensive to collect. Today, multiple principal investigator, multi-institution, and even multicountry studies are common, and such studies have resulted in unprecedented insights regarding human health. Researchers now need, or are even expected to share, data between different universities, across entities in different sectors (eg, universities, corporations, and nonprofits) and frequently across international borders. It also used to be that the scale of research was closely linked to the research methods. For instance, intervention studies were conducted with small numbers of participants in tightly controlled environments, and large-N surveys tended to collect data in ways that limited the possibility of individual identification and promoted easy anonymization. Today, expanding computational capabilities, social media, and broad research networks allow us to conduct an intervention study on Facebook with millions of participants [6], engage patients using mobile phone technology [7], study the whole genomes of thousands of individuals [8], or collect digital traces of human activity [9] at such granular levels that reidentification of individuals is possible if one possesses the right tools and expertise [10]. While traditional approaches to research require collecting only as much data as is necessary to test a hypothesis, data mining and other big data techniques derive their power from large data sets, where it may be impossible to determine, a priori, which variables will be of interest.

### A Static Regulatory Environment

In contrast to the evolving research practices landscape, procedures for research oversight have been markedly static. The Common Rule, which refers to a set of regulations that specify the procedures for establishing and operating IRBs, was adopted in 1991, and the Belmont Report and the Common Rule remain the primary sources for guiding review of human research. In 2011, recognizing that these regulations had not kept pace with the evolving human research enterprise, the US Department of Health and Human Services issued an Advance Notice of Proposed Rulemaking (ANPRM) aimed at “enhancing protections for research subjects and reducing burden, delay, and ambiguity for investigators” [11]. In 2015, the ANPRM transitioned to a Notice of Proposed Rulemaking (NPRM), the next step in the process to update federal regulations [12]. The NPRM updates include, for example, new consent requirements for biological specimens, use of a central IRB for multisite studies, and changes to procedures for determining exempt versus expedited study review categories. Use of a central IRB is particularly contentious, with concerns focusing on whether protection of participants may be compromised for the increased efficiency of a single IRB [13]. Regardless, in June 2016, the US National Institutes of Health published a policy requiring single IRB review for multisite studies [14]. While the NPRM reflects important and potentially promising activity toward IRB system improvements, many have questioned or objected outright to some of the proposed changes, and even supporters have suggested they are not ideal [15]. Most recently, a report by the National Academies of Science, Engineering, and Medicine Committee on Federal Research Regulations and Reporting Requirements issued a report criticizing the NPRM, citing that the proposed changes would be detrimental to advancing research [16]. This committee recommended that the US Congress authorize the presidential appointment of a national commission to examine and update the ethical and regulatory frameworks governing human research protections. Regardless of whether and to what extent the Common Rule or principles of the Belmont Report are revised, the extent to which IRBs can keep abreast of changes in the research landscape and be responsive to studies that leverage emerging technologies remains questionable at best.

### A Flawed Institutional Review Board System

There is increasing evidence that the IRB system is deeply and inherently flawed [17]. Lidz and colleagues captured the tip of the proverbial iceberg in their study of 20 IRB panels at 10 large medical institutions, where they documented 104 protocol reviews [18]. They found that IRBs consistently discussed the informed consent document, one of the Common Rule’s central mandates, and requested changes to the consent document in 88% of those cases. They also documented a disturbing observation, which was that other elements of the Common Rule (eg, data monitoring and protection of vulnerable populations) that are intended to promote research ethics were rarely discussed. Furthermore, in studies that exceeded minimal risk of harm, 21% of reviews did not address the inclusion of strategies to minimize risk. Likewise, they noted that 50% of reviews did not compare risks and benefits, and 60% of the protocols that excluded groups of potential research subjects without explicit justification were not discussed. They also found that critical review of the research design and methodology was not uncommon, and that IRBs often requested that investigators make changes to their research design, which is typically considered outside the purview and mission of IRBs.

Taken together, these observations call into question whether IRB members are sufficiently familiar with the standards intended to guide their review of research. Furthermore, while this is the case with respect to studies that leverage traditional research methods (eg, clinical trials), these concerns are magnified when the studies under review involve emerging technologies and nontraditional methods that the IRB was not originally designed to handle and that IRB members often do not understand. Examples of such studies are those that use smartphone capabilities to measure physical activity, social media to assess adverse drug reactions, or N-of-1 genome sequencing studies for diagnosis of rare disease. Such studies raise new and nuanced ethical issues regarding participant privacy, informed consent, and data security. Some of these novel methods also inadvertently include nonparticipants [19] or “bystanders” [20] in the research record, raising potential concerns that further challenge IRB processes.

## Methods

### How Might We Redesign the Institutional Review Board?

In light of these issues, in January 2015 we assembled a multidisciplinary group of 11 researchers and IRB professionals drawn from academic and research institutions in San Diego, California, to consider how we might reimagine and redesign human research protections for 21st century science. The half-day workshop was set up as a brainstorming session to generate ideas for addressing IRB challenges related to review of human studies, with a particular focus on studies that leverage emerging technologies and methods. The aim was not only to stimulate creative thinking about how the existing IRB structure and process could be modified to meet the often cited challenges of the current system, but also to generate ideas for exploring entirely new ways of evaluating research to ensure that research participants are informed and protected.

### Design Thinking

A central feature of this workshop was the use of design thinking strategies in the brainstorming process. Design thinking is a formal method for practical and creative resolution of problems [21] that emphasizes a phase during which the group or team focuses on generating as many ideas as possible using thoughtful prompts (eg, How might we advise as opposed to restrict? How might we simplify IRB review?). Design thinking is also considered particularly useful when the problem itself, in addition to the solution, may be unknown or ill defined at the outset of the problem-solving exercise.

### Workshop Description and Stages

Workshop participants included a facilitator (SK), a cofacilitator (CB), and 9 participants (the remaining authors). A high-level goal of the session was to generate ideas for how we might reimagine and ultimately redesign the human research protections system to foster the ethical conduct of research in the changing landscape of 21st century science.

The design thinking protocol consisted of 3 primary stages. During the first stage, we asked participants to brainstorm ideas using 6 categories as prompts: (1) settings, scenarios, and steps; (2) stakeholders and extreme users; (3) utopia and dystopia; (4) change levers; (5) change agents and obstacles; and (6) things to find out. During the second stage, we asked participants to consider the ideas generated in stage 1 and to complete the sentence “How might we...?” using the stage-1 ideas as prompts. A total of 22 “How might we…?” statements were generated (see Textbox 1). From the full list of “How might we…?” statements generated in stage 2, we asked participants to select 3 ideas that they were most interested in pursuing further.

In stage 3, participants were broken into groups based on overlapping interests to further discuss and expand on specific ideas. The 5 refined “How might we…?” statements that received the most votes were (1) How might we redesign the consent form and process? (2) How might we empower researchers to protect their participants? (3) How might we learn from our efforts to protect participants? (4) How might we make the IRB system more efficient? and (5) How might we help IRBs review new forms of research enabled by technological advances? The group discussions related to each of these ideas are presented below.

**“How might we…?” statements.**

- Start a learning health system experiment?
- Share all of our data?
- Prevent those interested in profit from taking advantage of those interested in science?
- Conduct bold experiments? (Incentivize and facilitate)
- Expedite institutional review board (IRB) review? (More appropriately classify)
- Make consent actually informed?
- Increase transparency of IRB processes and outcomes?
- Set up an appropriate surveillance system to monitor ethical violations?
- Simplify IRB review?
- Abolish IRBs?
- Reframe the IRB as a research partner rather than a research barrier?
- Increase confidence in anonymization?
- Create a learning system where IRB evolves along with research practices?
- Engage the public in research and in helping IRBs?
- Assess the true cost of the IRB system? (Direct and indirect; What are we not doing [that we should be] because of the current IRB process?)
- Collect more empirical research on the current state of the IRB?
- Create a movement around IRB?
- Influence current legislation wisely? (Start at the state level to guide national policy; eg, California Embryonic Stem Cell Research Oversight [ESCRO] committee)
- Seek an IRB waiver process?
- Include topic experts in IRB decision-making processes?
- Advise as opposed to restrict?
- Move from permission to forgiveness?

## Results

### Redesigning the Consent Form and Process

The ethical principle of respect for persons implies that individuals should be informed about and voluntarily consent to participate in research. How do we ensure that consent is actually informed? How do we ensure that research participants from diverse backgrounds truly understand research study risks and opportunities? In regard to the first question, one idea may be to establish mechanisms through which participants can provide real-time feedback about their experiences to researchers. These mechanisms could serve to collect empirical data regarding the clarity of consent forms and potential participants’ perceptions of risks and benefits. These data could inform and drive potential revisions to the consent form and other aspects of the research protocol. Relatedly, it is often the case that investigators write their consent forms to adhere to institutional templates, which may prompt the inclusion of content that is not relevant to or appropriate for a study. Thus, accurate and understandable descriptions of research should be encouraged in consent forms and processes, and inappropriate adherence to templates should be discouraged.

In addition, to make the informed consent process more accessible, one idea may be to think of the Creative Commons licenses [22] as a model. Similarly to the “three layers of licenses” used by Creative Commons, research studies could create three consent forms: one that contains all the legalese and scientific exposition; one in plain English that presents the facts; and a third that is simplified even further and presents risks in bullet point format. To make the process of obtaining consent culturally appropriate for underserved and underrepresented populations, community leaders, such as a *Promotor/a* in a Latino community, could be asked to help design the consent form and facilitate its use in ways that address community-specific concerns that researchers might not anticipate. Researchers could work with the community leader to help communicate these risks in a way that resonates with the community.

### Empowering Researchers to Protect Participants

It may be worthwhile to consider how to construct a system of human research protections that fosters the ethical conduct of research without relying on an institution like the IRB. How might we start anew and reimagine and redesign research oversight without the traditional IRB in mind? What would an alternative system look like? One idea is to place responsibility for participant protection on the researcher rather than on the IRB. Researchers intending to engage in human-participant research could produce a document that lays out plans and risks of the research. They could then offer those documents, along with an outline of the proposed consent process, for review by their peers. Peers would be researchers in the field of relevance for the research. These documents could be posted on the Web in the same way clinical trials are registered; not to get approval but to create a public record of the research. Peers who review the documents might be accredited with some type of certification in human research protections, although an open question would be what entity would design and provide such certification (and how such an entity would look different from a traditional IRB). Obtaining this certification and participating in this process could be incentivized for researchers by considering these activities to be professional service required for career advancement and academic promotion. In this scenario, responsibility for ethical conduct during the study would be shared by both the researchers and the peers who agreed that the plan would adequately protect participants.

To make it easier to create high-quality plans, researchers could consult a Web-based resource similar to Stack Overflow [23], a resource that software developers often use to obtain quick answers from experts about specific technical issues. With this resource, the median response time is 11 minutes [24], and the people responding are rated, which provides information pertaining to their credibility and expertise. Using this Web-based resource, within a few hours, researchers posing questions such as “How do I ensure that I won’t cause harm by asking this interview or survey question?” would receive answers from researchers who have been rated in terms of experience and expertise in human research protections. Elements of the plans could ultimately become like “protection modules” that could be swapped in and out of consent forms and research protocols, drawing attention to highly ranked modules. We note, however, that such a solution would require an active community with a critical mass of users, which may or may not be realistic depending on whether the IRB process ever became truly standardized. Importantly, if such a system were found to be feasible, it is an approach that could be coupled with a system that punishes offenders (see below).

### Reinforcement and Learning From Experience

This notion also begins with the premise that the burden to protect participants be shifted to the researcher rather than remain with an IRB or other regulatory body. How might we simultaneously reduce the bureaucratic burden of IRBs for researchers, particularly those conducting low-risk studies, and, at the same time, improve protection for research participants? In addition, how might we transform universities into learning ethics institutions that continuously improve their capacity to conduct ethical research [25]? One model for doing this could be the US Federal Aviation Administration’s Aviation Safety Reporting System [26]. Pilots who have a “bad” landing or make another safety-related error who self-report their mistake are spared from punishment, but those who do not report it themselves are penalized if someone elects to report [27]. Analogously, as an alternative to an IRB, in this system, researchers who create a protocol they believe to be safe, who then observe a harm during the research and who report that harm to their university or institution, present an opportunity for the research institution and community to learn how to prevent future harm. This expectation would be reinforced because, if the harm were to be reported by anyone else, including research staff or the research participant, the researcher would be sanctioned. That being said, there are clearly potential risks of supplanting the traditional IRB with a system entirely driven by researcher self-regulation. There could be conflicts between the researchers’ mandate to conduct studies and publish them and their mandate to protect participants, thus creating the opportunity for bias, the perception of bias, or, in extreme cases, maleficence. A system of researcher self-regulation would need to carefully consider and guard against these potential threats.

### Increasing Efficiency of the Institutional Review Board

We suggest that, in order to improve the IRB process, it is essential to understand its costs, both direct and indirect. How might we collect and analyze empirical data on costs of the IRB system? Obvious tangible costs associated with the IRB system include salaries of personnel, IRB fees, space and infrastructure costs, and fees paid for training, education, and accreditation. In addition, for researchers, costs include the amount of time required for study staff to prepare and process a study protocol through the IRB. Depending on the institution and the type of protocol, IRB submissions can be extremely time intensive to prepare, which is an opportunity cost in terms of other ways in which that time could be spent. For research participants, costs include the time and cognitive effort needed to understand increasingly complex and bureaucratic consent forms. There are also less-tangible costs related to the broader public health caused by unnecessary delays to research imposed by IRBs.

One idea to increase efficiency may be to use the “Cooperative Research” process (see 45 CFR 46.114 [28]) to reduce the multiple IRB review of multisite studies and to use the “exempt” category to a greater degree, as it was intended. The exempt category is frequently appropriate for the vast majority of social and behavioral science studies, yet it is underused, which leads to delays in review and approval [29] and, thus, wasted resources. In addition, IRBs could take care to ensure that the process of review for exempt studies is reasonable and truly reflects their low-risk nature. Interestingly, exempt research, according to US federal regulations, does not need to be verified or reviewed by IRB staff. If institutions permitted, determining exempt status could be made the responsibility of the researcher. Overall, the idea that the bureaucracy of the IRB creates a significant burden to the research enterprise while producing unclear or intangible benefits to research participants is consistent with the purported rationale cited for the development of the proposed revisions to the Common Rule in the form of the NPRM [11] discussed above. We suggest that the IRB may benefit from an analysis of costs and benefits of its own activities, much like it does with the studies it oversees.

### Review of Research That Leverages Technological Advances

New forms of research enabled by technological advances in information technology, data science, and other fields appear to be particularly challenging to IRBs. How might we develop resources that would facilitate appropriate review of 21st century science? The California Institute for Regenerative Medicine (CIRM) research oversight process could serve as a model. In CIRM 1.0, a committee separate from the IRB called the Embryonic Stem Cell Research Oversight (ESCRO) committee was formed to review stem cell research. Recognizing that few IRB members would have sufficient expertise to provide a meaningful review, the ESCRO committee, which is composed of scientists and a community representative, serves in an advisory capacity to the IRB. Such a model could be replicated for studies using emerging technologies about which IRBs may be similarly unfamiliar to ensure that experts are involved in the review.

For example, mobile, visual imaging, pervasive sensing, and geolocation tracking technologies present new ethical and regulatory challenges [20]. For instance, visual imaging using wearable sensors have made it possible for researchers to measure physical activity, diet, travel, and the settings in which these behaviors occur using a first-person point-of-view wearable camera. Given the increasing interest in these methods for studying behavior “in the wild,” we anticipate increased research using visual methods, which raises privacy concerns and issues related to the rights of bystanders. Likewise, with wearable sensors, mobile phone transmission, and analytics in the cloud, health information can be captured continuously in real time. Location tracking technologies provide spatial data and the opportunity for assessing the context in which behavior is occurring, as well as identifying underlying spatial relationships such as clustering or transmission pathways. These data are fine grained and specific down to the exact longitude and latitude at a given point in time. Standards for how these data are transmitted, stored, and shared are necessary, since the introduction of the US Health Insurance Portability and Accountability Act, in most cases (at least at present), does not apply. A virtual network composed of researchers, technologists, and bioinformatics experts may prove to be a workable solution to augment or replace the traditional IRB review process resulting in an informed and meaningful human protections review of 21st century science.

## Discussion

In this paper we imagine, and offer some ideas for the design of, a progressive, responsive, and nimble human research protections system. By encouraging broad and innovative ideas, the design thinking method not only opens up new avenues for exploration, but also provides clarity about some of the shortcomings of our current IRB system. The workshop described here aimed to stimulate creative thinking about how the existing IRB structure could be improved, while also generating ideas for entirely new ways of protecting research participants. Clearly, some of the ideas presented here are more feasible than others. For example, it may be more realistic to encourage IRBs to exploit the current regulations and use the “exempt” category more appropriately and frequently. Alternatively, imagining a completely different review process that would replace the traditional IRB entirely may be less acceptable and would likely create new problems. If the proposed NPRM is adopted, we note that some research may be excluded from the traditional IRB review yet may benefit from an “ethics consultation” process to avoid making mistakes that an IRB may have detected. It would be valuable to estimate the cost savings realized by implementing the new practices that reduce the burden on both IRBs and researchers without compromising human research protections. Clearly, further debate by stakeholders is necessary to develop these and other ideas into concrete recommendations to advance applied human research ethics.

Sometimes, systems are so entrenched in ways of doing things that change from within is not possible and disruptive external approaches are required (eg, Uber as an alternative to transportation via taxis, or specialty charter schools as an alternative to traditional public schools). The IRB may be a system in need of disruption. Using the design thinking method fostered the development of “outside the box” ideas that may improve research participant protections and the IRB structure. As such, we initiated the exercise described in this paper and seek to share the process and results with the greater research community. The IRB system will need to be updated or possibly even reinvented in order to be responsive to technological advances of recent decades that have enabled new forms of research. These advances have created challenges to our current system that the NPRM will not likely solve. Pilot research programs that test-drive the reform ideas presented here, or perhaps other ideas, would be worthwhile and informative as the research community considers how to intervene and make healthy what many believe is an ailing human research protections system.

## Abbreviations

ANPRM: Advance Notice of Proposed Rulemaking
CIRM: California Institute for Regenerative Medicine
ESCRO: Embryonic Stem Cell Research Oversight
IRB: institutional review board
NPRM: Notice of Proposed Rulemaking
